# Role of the PP2A Pathway in Cholangiocarcinoma: State of the Art and Future Perspectives

**DOI:** 10.3390/cancers14215422

**Published:** 2022-11-03

**Authors:** Ion Cristóbal, Angela Lamarca

**Affiliations:** 1 Cancer Unit for Research on Novel Therapeutic Targets, Oncohealth Institute, IIS-Fundación Jiménez Díaz-UAM, 28040 Madrid, Spain; 2 Medical Oncology Department, Oncohealth Institute, IIS-Fundación Jiménez Díaz-UAM, 28040 Madrid, Spain

## Introduction

Cholangiocarcinoma represents a heterogeneous disease at both a clinical and molecular level. The fact that it is usually diagnosed at advanced (non-resectable) stages significantly contributes to its marked poor prognosis. Despite treatment advances in the last decade, patient outcomes are still very poor, with the 5-year survival rate below 20% [1]. Only 20–30% of cholangiocarcinoma will be diagnosed at a resectable stage; despite surgery and adjuvant treatment, the relapse rate remains high [2]. Thus, the majority of patients diagnosed with cholangiocarcinoma will, at some point in their journey, receive treatment with a palliative aim for advanced or recurrent disease.

Palliative treatment options for cholangiocarcinoma are evolving. The development of targeted therapies has dramatically changed the scenario, but these are only suitable for a small proportion of patients [3]. For all patients without targetable alteration, the standard first-line treatment relies on cytotoxic chemotherapy in the form of cisplatin and gemcitabine, with second-line treatment strategies of 5-fluorouracil (5-FU) and oxaliplatin (FOLFOX), and nanoliposomal irinotecan combined with 5-FU as potential options [4]. Recently, the addition of durvalumab to cisplatin and gemcitabine has shown improved outcomes in the first-line setting [5]. Despite these treatment options, response rate and survival remain poor. It is imperative to improve our knowledge around the molecular mechanisms that govern cholangiocarcinoma progression, in order to develop novel predictive biomarkers and alternative therapeutic strategies that improve the current unfavorable patient outcomes. In this regard, to date, the PP2A pathway has been poorly investigated in cholangiocarcinoma in comparison with other central pathways, but preliminary evidence in the literature suggests that it could be of high relevance in this disease.

PP2A is a well-known tumor suppressor that regulates a wide variety of signaling pathways and cellular processes. Therefore, PP2A inhibition has been described as a common alteration in many tumor types, and overexpression of its endogenous inhibitors SET and CIP2A has been described as key molecular alterations that contribute to inactivate this phosphatase in human cancer [6]. In recent years, the PP2A pathway has emerged as a druggable tumor suppressor with important roles as a regulator of treatment efficacy in many tumor models [7]. This issue could also be of interest in cholangiocarcinoma, since the PP2A pathway has been demonstrated to modulate tumor sensitivity to the chemotherapeutic agents used in standard treatments such as gemcitabine, cisplatin, 5-FU, oxaliplatin and irinotecan. Thus, PP2A has been shown to enhance sensitivity to gemcitabine, negatively regulating AKT through its dephosphorylation in pancreatic cancer cells [8]. Moreover, several studies have demonstrated the role of the PP2A pathway-enhancing response to 5-FU and overcoming resistance to this compound [9,10,11]. However, it seems that certain PP2A complexes such as those harboring the B56delta subunit could have opposite functions [12], which is concordant with a previously described dual function of some specific PP2A subunits in cancer [13]. The importance of PP2A as a key modulator of cisplatin sensitivity has also been described in different tumor types such as lung cancer [14] and oral squamous cell carcinoma [15], and FTY720-induced PP2A activation has shown synergistic effects with cisplatin in hepatoblastoma cells [16]. Finally, PP2A has been reported to regulate the response of lung cancer cells to irinotecan treatment [17].

In concordance with the expected tumor suppressor role of PP2A in cholangiocarcinoma, the work by Lu and colleagues [18] showed that the PP2A activator FTY720 inhibited proliferation, invasiveness and epithelial-to-mesenchymal transition, as well as promoted apoptosis and cell cycle arrest in vitro and affected tumor growth and metastasis in vivo. Of note, it has also been reported that cantharidin induces antitumor effects in cholangiocarcinoma cell lines that are partially dependent on its PP2A inhibitory activity, thereby resulting in activation of the IKKα/IκBα/NF-κB pathway [19]. The authors claimed that cantharidin is a specific PP2A inhibitor. However, its effects on PP1 at the micromolar level (similar to PP2A) and, more importantly, its inhibitory effects on PP5 at the nanomolar level could modulate these reported effects, and their contribution should be clarified [20]. In fact, PP5 has been demonstrated to play oncogenic functions in cholangiocarcinoma cell lines in vitro and in a xenograft model derived from the human liver bile duct carcinoma cell line HuCCT1 in vivo, at least in part directly binding and regulating AMPK activation [21]. These considerations are further supported by recent data in the literature demonstrating the opposite functions of PP2A and PP5 in human cancer in the regulation of key pathways such as MAPK signaling [22].

Furthermore, microcystin-leucine-arginine (MC-LR) promoted the survival of cholangiocarcinoma cells via PP2A inhibition and ulterior activation of the ERK/MEK signaling axis through a direct positive regulation of the PP2A inhibitor SET at the transcriptional level. Moreover, the content level of MC-LR was identified as an independent predictive factor of poor prognosis in a cohort of 58 intrahepatic cholangiocarcinoma patients [23]. In addition, SET has been reported to induce 5-FU and oxaliplatin resistance in colorectal cancer [24,25], and has been described as a key mediator of the acquisition of a cisplatin resistance phenotype in lung cancer [26]. Notably, several data in the literature regarding the involvement of CIP2A in cholangiocarcinoma further highlight the potential relevance of the PP2A pathway in this disease. CIP2A is a well-known endogenous PP2A inhibitor that plays oncogenic functions promoting the proliferation, migration, drug resistance, stemness or invasiveness abilities of tumor cells. CIP2A overexpression has been largely reported as a common event in human cancer and represents a key molecular mechanism to inactivate PP2A [27]. Moreover, this alteration has shown clinical impact as a biomarker of poor outcome in many tumor types, including cholangiocarcinoma. In fact, Xu and colleagues [28] analyzed a cohort of 57 patients with cholangiocarcinoma as well as a set of 23 tumor adjacent normal bile duct samples. They found that CIP2A expression was higher in tumor samples than in normal tissues and represents an alteration that independently predicted shorter overall survival, suggesting its potential usefulness as a poor prognostic marker in this disease. Moreover, CIP2A downregulation has been described to increase sensitivity to gemcitabine in pancreatic cells [29]. The fact that gemcitabine plus cisplatin is widely used in a standard first-line chemotherapy regimen in patients with unresectable cholangiocarcinoma [30] suggests a potential relevance of CIP2A as a novel molecular target in this disease. Of note, these findings are further supported by the fact that the long intergenic non-coding RNA 00665 (LINC00665), which encodes a micropeptide CIP2A-BP [31], has been reported to promote the gemcitabine resistance of cholangiocarcinoma cells [32]. Moreover, CIP2A downregulation has been found to increase the sensitivity of lung cancer cells to cisplatin [33] and SN-38 (an active metabolite of irinotecan) [34], suggesting that it could also have relevance in re-sensitizing cholangiocarcinoma cells to these treatments. Although the reported effects of CIP2A and SET on gemcitabine, cisplatin, 5-FU, oxaliplatin and irinotecan are probably due to their role as potent endogenous PP2A inhibitors, it would be very interesting to evaluate whether they can also activate PP2A-independent molecular mechanisms to regulate efficacy in these compounds.

In conclusion, a significant amount of evidence in the literature suggests that the PP2A pathway could be of high relevance in cholangiocarcinoma. The endogenous PP2A inhibitors CIP2A and SET emerge as molecular target candidates in this disease, and the use of PP2A activators to overcome resistance and restore sensitivity to current standard chemotherapy regimens should also be experimentally investigated in forthcoming studies. Whether these strategies could also enhance the activity of currently used chemotherapy options could also be hypothesized. Altogether, the PP2A pathway shows potential usefulness for improving the clinical management of this disease, improving response rates to current treatments as well as providing clinicians with novel predictive biomarkers of therapy response and outcome that need clarification.
